# Multimodal automatic assessment of acute pain through facial videos and heart rate signals utilizing transformer-based architectures

**DOI:** 10.3389/fpain.2024.1372814

**Published:** 2024-03-27

**Authors:** Stefanos Gkikas, Nikolaos S. Tachos, Stelios Andreadis, Vasileios C. Pezoulas, Dimitrios Zaridis, George Gkois, Anastasia Matonaki, Thanos G. Stavropoulos, Dimitrios I. Fotiadis

**Affiliations:** ^1^ Computational BioMedicine Laboratory (CBML), Institute of Computer Science, Foundation for Research and Technology – Hellas (FORTH), Heraklion, Greece; ^2^ Department of Electrical & Computer Engineering, Hellenic Mediterranean University, Heraklion, Greece; ^3^ Biomedical Research Institute, Foundation for Research and Technology – Hellas (FORTH), Ioannina, Greece; ^4^ Unit of Medical Technology and Intelligent Information Systems, Department of Materials Science and Engineering, University of Ioannina, Ioannina, Greece; ^5^ Pfizer Center for Digital Innovation, Thessaloniki, Greece

**Keywords:** pain recognition, deep learning, vision transformer, ECG, data fusion

## Abstract

Accurate and objective pain evaluation is crucial in developing effective pain management protocols, aiming to alleviate distress and prevent patients from experiencing decreased functionality. A multimodal automatic assessment framework for acute pain utilizing video and heart rate signals is introduced in this study. The proposed framework comprises four pivotal modules: the *Spatial Module*, responsible for extracting embeddings from videos; the *Heart Rate Encoder*, tasked with mapping heart rate signals into a higher dimensional space; the *AugmNet*, designed to create learning-based augmentations in the latent space; and the *Temporal Module*, which utilizes the extracted video and heart rate embeddings for the final assessment. The *Spatial-Module* undergoes pre-training on a two-stage strategy: first, with a face recognition objective learning universal facial features, and second, with an emotion recognition objective in a multitask learning approach, enabling the extraction of high-quality embeddings for the automatic pain assessment. Experiments with the facial videos and heart rate extracted from electrocardiograms of the *BioVid* database, along with a direct comparison to 29 studies, demonstrate state-of-the-art performances in unimodal and multimodal settings, maintaining high efficiency. Within the multimodal context, 82.74% and 39.77% accuracy were achieved for the binary and multi-level pain classification task, respectively, utilizing 9.62 million parameters for the entire framework.

## 1 Introduction

Pain, as defined by Williams and Craig (1), is a “*distressing experience associated with actual or potential tissue damage with sensory, emotional, cognitive and social components*.” Biologically, pain is an undesirable sensation originating from the peripheral nervous system. Its fundamental function is to engage sensory neurons, notifying the organism of potential harm and playing a vital role in recognizing and responding to threats (2). The principal categories of pain are acute and chronic, primarily differentiated by the duration of the sensation. Acute pain persists for less than twelve weeks. It is often accompanied by observable physiological damage, while chronic persists for over twelve weeks or exceeding the anticipated injury recovery period (3). Acute pain arises from injury, surgery, illness, trauma, or painful medical procedures and usually disappears whenever the underlying cause is treated or healed. However, without resolution, it can transition into a chronic condition, lasting beyond the initial acute phase. Postoperative pain, a facet of acute pain, arises specifically after surgical interventions and is a significant concern for both patients and healthcare providers, emphasizing the need for effective pain management strategies to facilitate recovery and prevent chronic pain development (4). Chronic pain exhibits various forms concerning the temporal dimension, such as chronic-recurrent (e.g., migraine headache) or chronic-continuous (e.g., low back pain) (5). Pain is a prevalent and diverse condition (6). According to the Global Burden of Disease (GBD) study, pain stands as the leading cause of years lived with disability (YLD) (7). The impact of pain extends beyond individuals to society, posing clinical, economic, and social challenges (7). Beyond the direct consequences on a patient’s life, pain is associated with various adverse effects, such as opioid use, drug overuse, addiction, compromised social relationships, and psychological disorders (8).

Effective pain assessment is essential for early diagnosis, monitoring the progression of the underlying disease, and evaluation of therapy outcomes, especially in managing chronic pain (9). This has led to the nursing literature referring to pain as “*the fifth vital sign*” (10). Objectively measuring pain is imperative for providing suitable care, especially for vulnerable populations unable to directly communicate their pain experiences, such as infants, young children, individuals with mental health conditions, and the elderly. Various methodologies are employed to assess pain, encompassing self-reporting, considered the gold standard for evaluating the presence and intensity of pain utilizing rating scales and questionnaires. Also, behavioral indicators, including facial expressions (e.g., grimacing, open mouth, or raised eyebrows), vocalizations (e.g., crying, moaning, or screaming), and bodily movements (including posture or signs of tension), serve as critical markers (11). Moreover, physiological measures, including electrocardiography, electromyography, skin conductance responses, and respiration rate, offer valuable insights into the physiological manifestations of pain (9).

Caregivers or family members typically rely on observing behavioral or physiological responses to infer the presence or absence of pain in patients (9). Despite its importance, pain assessment remains a formidable challenge for clinicians (12), particularly when dealing with nonverbal patients (13)—the elderly present additional challenges due to diminished expressive abilities or unwillingness to communicate (14). Moreover, extensive research (15) highlights significant variations in pain manifestation across genders and ages, underscoring the complexity of the assessment process. There are additional complexities in pain assessment; heightened workload and fatigue among nursing staff have been reported due to challenges of patient monitoring (16). Concerns persist regarding the objectivity and accuracy of observations, where inadequately trained or biased observers may find it challenging to assess a patient’s pain appropriately (17). Furthermore, variations in interpreting behaviors may arise even among trained observers (11). Social and interpersonal dynamics also significantly impact the pain assessment process, influencing the judgment of evaluators and the outward expression of pain by those being evaluated (18). In several cases, patients may alter their behavior in the presence of an observer (19), or it is difficult to express the pain through scales and measurements (20). The self-report, although pain is fundamentally a subjective experience, a one-dimensional pain score inadequately evaluates this complex phenomenon, resulting in insufficient pain treatment (21).

Due to the challenges mentioned above, substantial research is dedicated to advancing automatic pain identification systems, aiming to discern the presence and intensity of pain by analyzing physiological and behavioral responses. In recent years, researchers in artificial intelligence (AI) have dedicated their efforts to developing models and algorithms to imbue machines with cognitive capabilities, explicitly emphasizing the nuanced task of identifying complex emotions and affective states, including the intricate domain of pain. The advent of deep learning methods has further driven the exploration of these approaches for automated pain assessment, signifying a critical stride toward more accurate and efficient methodologies in this domain (9). Numerous studies have underscored the potential of automated systems leveraging behavioral or physiological pain assessment modalities (22). Sario et al. (23) assert the feasibility of accurately detecting and quantifying pain through facial expressions, showcasing their potential as a valuable tool in clinical practice. The integration of multimodal sensing is particularly promising, suggesting enhanced accuracy in pain monitoring systems (11). Considering the temporal dimension of these signals has been associated with improved and more precise pain assessment (9). Another critical aspect of pain monitoring systems revolves around the utility of wearable devices that record biopotentials for estimating pain levels. A limited number of studies have explored the employment of mainstream wearable technology for data collection, potentially due to a preference among researchers for more expensive, highly accurate medical equipment. According to Leroux et al. (21), “*The challenge is not whether wearable devices will provide useful clinical information but rather when we will start to use them in practice to improve the field of pain.*” Furthermore, Claret et al. (24) explore the potential use of cardiac signals acquired from wearable sensors for automatic emotion recognition, affirming the viability of such an approach.

This study presents a proof of concept for an automatic pain assessment framework integrating facial video data from an RGB camera with heart rate signals. The framework is based on four main components: the *Spatial Module*, creating embeddings from video data; the *Heart Rate Encoder*, transforming heart rate signals into embedding representations; the *AugmNet*, generating augmentations within the latent space through learning-based methods; and the *Temporal Module*, which leverages the video and heart rate embeddings for the final pain assessment. Our main contributions are: (1) the assessment of the effectiveness and the limitations of using video and heart rate as standalone modalities in a unimodal manner, (2) the examination of the efficacy of combining behavioral (video) and physiological (heart rate) markers, driven by the need to address challenges arising from their reliance on different sensing technologies and information representation, and finally, (3) the analysis of the recently introduced transformer-based architectures, focusing not only on their performance but also their efficiency. Contrary to other related studies focusing on raw cardiac signals, such as electrocardiography (ECG) (25) and photoplethysmography (PPG) (26), or extracting various features (27), including heart rate (28), this study highlights the practical value of heart rate as an isolated input. It can easily be acquired with wearables, requiring no additional computation stages, making it a potentially important information source in an automatic pain assessment process.

## 2 Related work

Extensive research has been dedicated to estimating human pain levels, employing individual input modalities, or exploring the integration of various information channels in a multimodal fashion. Leveraging publicly available pain datasets, which encompass behavioral and physiological modalities as those found in the *BioVid Heat Pain Database* (29), researchers have introduced and proposed a wide array of methods. Each approach carries distinct merits and drawbacks, encompassing complexity considerations, computational cost, and performance. These factors are critical for practical application in real-life scenarios, such as clinical settings.

Various innovative approaches have emerged to estimate pain levels from video data. Werner et al. (30) introduced an optical flow method that tracks facial points to capture changes in facial expressions across frame sequences. Focusing on the dynamic nature of pain led to the development of long short-term memory networks with sparse coding (SLSTM) (31). Tavakolian et al. (32) proposed 3D convolutional neural networks (CNNs) with varying temporal depths to capture short-, mid-, and long-range facial expressions. A 3D CNN with self-attention structures to enhance the significance of specific input dimensions was presented in (33). Two strategies were employed to exploit the video’s temporal dimension–encoding frames into motion history and optical flow images, followed by a framework incorporating a CNN and a bidirectional LSTM (biLSTM) (34). Videos were encoded into single RGB images using statistical spatiotemporal distillation (SSD) and trained a Siamese network in a self-supervised setting (35). Werner et al. (36) adopted a domain-specific feature approach, proposing facial action markers classified by a deep random forest (RF) classifier. They have also suggested a method of 3D distance computation among facial points, yielding comparable results. Patania et al. (37) utilized deep graph neural network (GNN) architectures and dense maps of fiducial points to detect pain, while (38) presented a multi-task framework combining person identity recognition and pain level estimation, utilizing a CNN with an autoencoder attention module. Huang et al. (39) detected facial regions and employed a multi-stream CNN for feature extraction, consisting of four sub-CNNs, one for each facial region. An interesting element of their framework was assigning learned weights to extracted features, offering attention based on the varied contribution of each facial region to pain expression. In a subsequent study (40), the authors recognized that specific frames vividly exhibit pain expressions in a video sequence. Consequently, they developed a novel framework with attention saliency maps using CNNs, gated recurrent units (GRUs), and learned weights associated with each frame’s contribution to the final pain intensity estimation. The study highlights the potential for compelling performance by exploiting dynamic and salient features, while in (41), an efficient transformer-based model achieving compelling results was proposed.

Multiple studies have also explored unimodal approaches focusing on the cardiac signal of electrocardiograms for the recognition of acute pain. Martinez and Picard (27) devised a recurrent neural network (RNN) and trained it on the extracted R peaks and inter-beat intervals from ECG signals. Thiam et al. (25) employed deep 1D CNN, incorporating ECG, electrocardiogram (EMG), and galvanic skin response (GSR) signals. Their research covered both unimodal approaches and multimodal fusion techniques. Notably (33), proposed a framework to derive pseudo heart rate information from videos using a 3D CNN, achieving high performance in binary and multiclass classification settings. In (42), heart rate variability features were extracted, and a random forest classifier yielded significant results in pain detection. Various features were calculated from inter-beat intervals, including heart rate, demonstrating notable outcomes (28). In a follow-up study (43), the development of multi-task fully connected neural networks led to a significant increase in performance.

Given the multidimensional nature of pain, a promising route involves integrating modalities within a multimodal system. The combination of diverse information sources has the potential to enhance both specificity and sensitivity for the pain assessment. Individual modalities demonstrate satisfactory predictive performances, but their fusion generally results in improved outcomes (22). Additionally, the use of cues from various channels could prove not only beneficial but also essential, particularly in clinical settings where, for various reasons, a modality may be inaccessible (e.g., the patient rotates, and facial visibility is obscured). Leveraging diverse features derived from both video and biomedical sources, including facial expression, head movement, GSR, EMG, and ECG, and employing various fusion methods, demonstrated highly promising outcomes (42). Multiple biopotential features were derived from ECG, EMG, and GSR, along with facial expressions and head pose features (44), while (45) focused solely on biosignals (again ECG, EMG, and GSR). In (46), three aforementioned biosignals were utilized, and combinations of handcrafted and learned features extracted from a biLSTM model were explored. Initially, the minimum relevance method (MRMR) was applied to reduce the number of features, yielding promising results. The study outlined in (47) utilized deep denoising convolutional autoencoders (DDCAE) to compute a latent representation for each biopotential, (i.e., ECG, EMG, GSR), followed by a weighting stage before the classification process presented promising results. Huang et al. (33) employed a 3D CNN and probabilistically combined computed facial features and pseudo heart rate information from the vision modality, achieving state-of-the-art performances.

This study aims to integrate behavioral and physiological modalities in a multimodal manner, combining facial videos with heart rate extracted from ECG signals. The proposed approach is the first to leverage videos and heart rate as the sole cardiac feature, resulting in high performances with minimal framework parameters.

## 3 Methodology

The preprocessing methods for video and ECG, the proposed framework design, the developed augmentation methods, and the implementation details of the pretraining process are described in this section.

### 3.1 Preprocessing

Prior to entering data into the framework for pain assessment, preparatory steps were taken to ensure the modalities were appropriately processed. Specifically, since raw ECG data is the input for the cardiac signal, computing heart rate becomes a crucial step. One of our primary objectives is to explore heart rate as the sole feature. This intention is motivated by its advantages, such as being readily obtainable from wearables, making it both cost-effective and easily accessible, thereby establishing it as a conceivably important feature for automatic pain assessment.

#### 3.1.1 Video preprocessing

Video preprocessing involved face detection to isolate the facial region. We employed the MTCNN face detector (48), which utilizes multitask cascaded convolutional neural networks for predicting face and landmark location. We mention that the prediction of landmarks is necessary since they enable face alignment. However, it was observed that the face alignment diminishes the expression through head movement, a behavioral manifestation of the pain. Therefore, face alignment was excluded from the proposed pipeline. In addition, it is important to note that the resolution of frames after the face detection process was set at 448×448 pixels.

#### 3.1.2 ECG preprocessing & analysis

**Figure 1:**
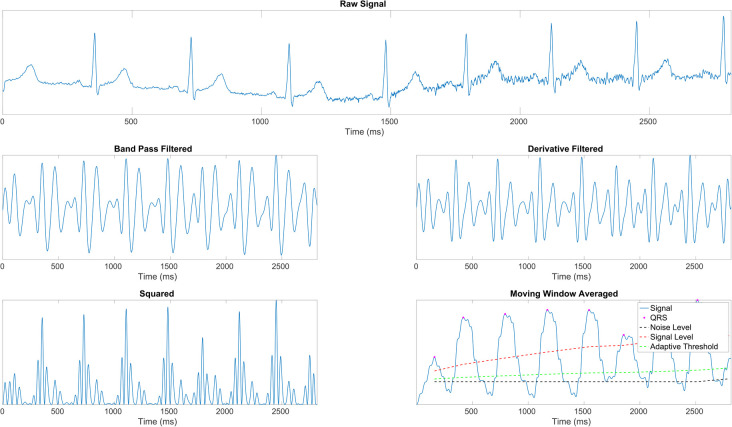
ECG signal preprocessing stages (43). **(1st row)** Raw ECG signal. **(2nd row, left)** Signal after band-pass filtering (BPF) to isolate the frequency range of interest. **(2nd row, right)** Signal post-derivative filtering to highlight the QRS complex. **(3rd row, left)** Squared signal to accentuate dominant peaks. **(3rd row, right)** Moving window average applied to the squared signal, illustrating the final signal (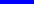) with identified R peaks (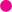), noise level (– –), signal level (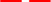), and adaptive thresholding (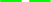).

We employ the Pan-Tompkins Algorithm (49) to detect the QRS complex, the most distinct wave complex in an ECG signal. The algorithm involves two stages: preprocessing and decision-making. Preprocessing addresses noise removal, artifact elimination, signal smoothing, and QRS slope enhancement. The decision-making phase covers initial QRS detection using adaptive thresholds, a retrospective search for missed QRS complexes, and a procedure for T wave discrimination. Figure 1 illustrates the preprocessing steps applied to raw ECG data. After the precise identification of R waves, the estimation of inter-beat intervals (IBIs) was undertaken, and the extraction of the most relevant features followed. Precisely, the mean of IBIs was calculated as:

(1) $$\mu =\frac{1}{n}\sum_{i=1}^{n}(RR_{i+1}-RR_{i}),\;$$

where n is the total number of IBIs, and $RR_{i}$ represents consecutive R time points. Subsequently, the following calculation was performed to determine the heart rate:

(2) $$HR=\frac{60\cdot FS}{\mu },\;$$

where FS denotes the sampling frequency of the ECG recording.

### 3.2 Framework architecture

**Figure 2:**
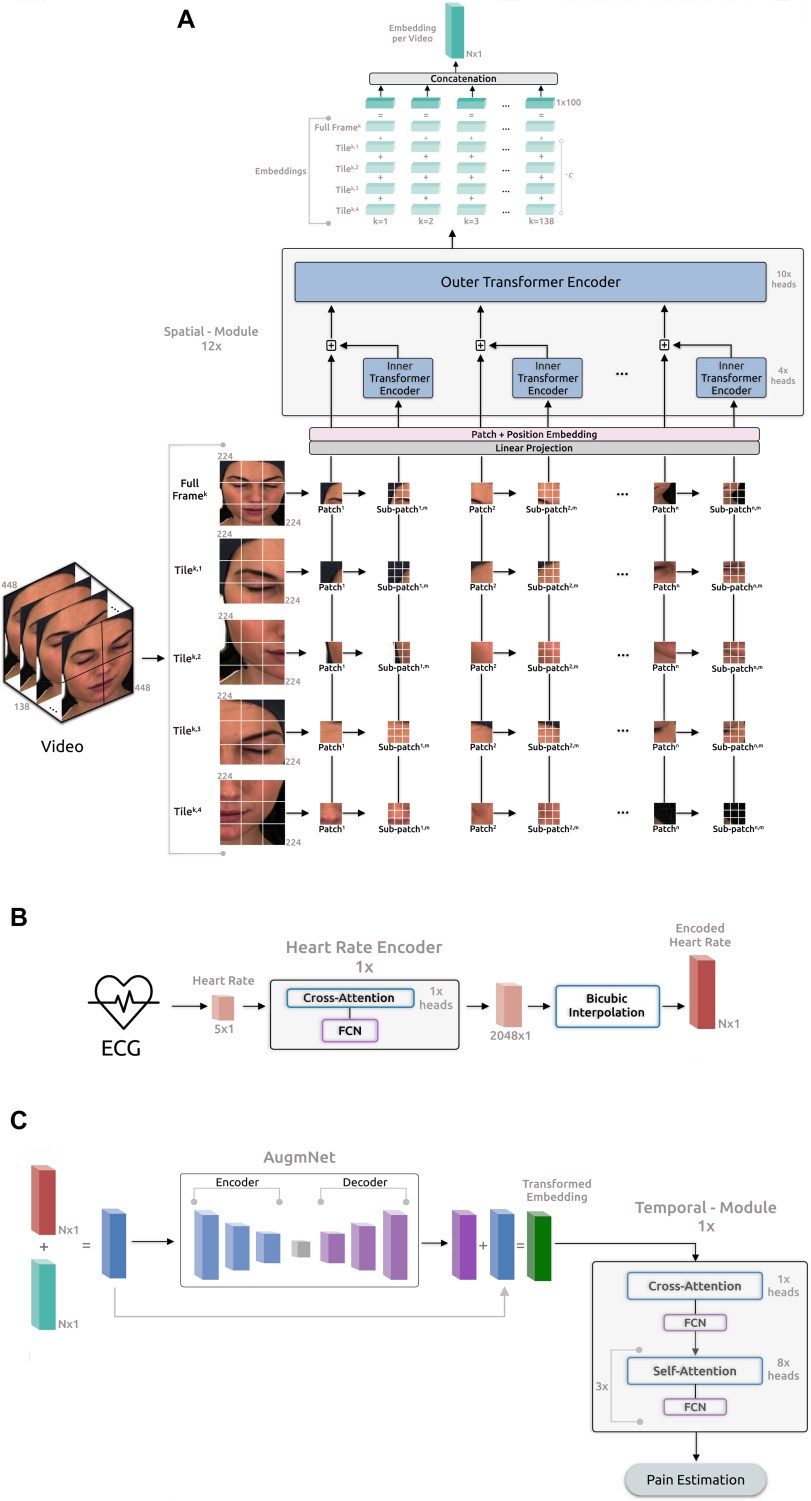
Overview of the proposed framework for automatic pain assessment. (**A**) Video analysis pipeline. (**B**) ECG analysis pipeline. (**C**) Fusion analysis pipeline.

The proposed framework (Figure 2) comprises four main components: the *Spatial-Module* extracting embeddings from the video, the *Heart Rate Encoder* mapping the heart rate signal into a higher dimensional space, the *AugmNet* creating augmentations in the latent space, and the *Temporal-Module* responsible for the final pain assessment.

#### 3.2.1 Spatial-module

The architecture of this module is based on the principles outlined in *“Transformer in Transformer*” as proposed by (50). The initial video frame has a resolution of 448×448 pixels and is segmented into 4 tiles (quadrants), each containing 224×224 pixels. The utilization of the tiling procedure, capitalizing on the original frame resolution, was inspired by the literature on satellite imaging analysis, where similar pipelines are applied. We leverage the 4 tiles and the original full frame in our proposed pipeline, resizing the latter to 224×224 pixels. Consequently, each video frame corresponds to a total of 5 images, $F^{k}=[F^{k,1},\;F^{k,2},\;\ldots F^{k,t}]$, where k is the individual frame number, and t is the tile number, including the resized full frame. Afterward, each tile is initially divided into n patches, denoted as $F^{k,t}=[F^{k,t,1},\;F^{k,t,2},\;\ldots F^{k,t,n}]\in R^{n\times p\times p\times 3}$, where p×p represents the resolution of each patch (16×16), and 3 indicates the number of color channels. Subsequently, these patches undergo further division into m sub-patches, enabling the model to capture the image’s global and local feature representations. Consequently, each input tile of a frame is converted into a sequence of patches and sub-patches, $F^{k,t}=[F^{k,t,n,1},\;F^{k,t,n,2}\cdots ,\;F^{k,t,n,m}]$. Therefore, each input video frame is represented as:

(3) $$F^{k}\to \left[F^{k,t,n,m}|t\in [1,\;5],\;\;n\in [1,\;196],\;\;m\in [1,\;16]\right],\;$$

where $F^{k,t,n,m}\in R^{s\times s\times 3}$ denotes the m-th sub-patch within the n-th patch of the t-th tile in the k-th frame of each video. The resolution of each sub-patch is s×s, specifically 4×4. Each frame comprises 5 image representations, each encompassing 196 patches, with each one of these patches containing 16 sub-patches. Subsequently, the patches and sub-patches undergo linear projection, resulting in embeddings Z and Y. The succeeding step involves position embedding to preserve spatial information for each patch. This process employs 1D learnable position encoding, assigning the position encodings to each patch:

(4) $$Z_{0}\leftarrow Z_{0}+E_{\mathrm{patch}},\;$$

where $E_{\mathrm{patch}}$ represents the position encoding. Likewise, individual position encodings are added for each sub-patch within a patch:

(5) $$Y_{0}^{i}\leftarrow Y_{0}^{i}+E_{\mathrm{sub}-\mathrm{patch}},\;$$

where $E_{\mathrm{sub}-\mathrm{patch}}$ represents the positional encodings for sub-patches, and i=1,2,…,m is the index of a sub-patch within a patch. The sub-patches undergo processing in the *Inner Encoder*, comprising 4 self-attention heads (51), employing dot product attention and represented as:

(6) $$Attention(Q,\;K,\;V)=softmax\left(\frac{QK^{T}}{\sqrt{d_{k}}}V\right).$$

The output embedding from the *Inner Encoder* is incorporated into the patch embedding, leading the combined representation to the subsequent *Outer Encoder* process. The *Outer Encoder* essentially mirrors the *Inner Encoder* structure, featuring 10 self-attention heads. The entire *Spatial-Module* comprises 12 parallel blocks, collectively producing embeddings with a dimensionality of d=100.

For each input video frame, 5 distinct output embeddings are generated, each with a dimensionality 100. These embeddings are then added together, creating a final embedding representation for the frame:

(7) $$D=d_{\mathrm{FullFrame}}+(d_{{\mathrm{Tile}}^{1}}+d_{{\mathrm{Tile}}^{2}}+d_{{\mathrm{Tile}}^{3}}+d_{{\mathrm{Tile}}^{4}})\cdot c,\;\;D\in R^{100},\;$$

where c is a constant applied exclusively to the embeddings of the tiles to retain only a proportion of the original information encapsulated in these embeddings. Next, the embedding representation D for each frame is concatenated with the embeddings of the remaining frames. This process creates a final embedding representation for the entire video:

(8) $$V_{D}=[D_{1}\|D_{2}\|\ldots \|D_{\;f}],\;\;V_{D}\in R^{N},\;$$

where f denotes the number of frames in the video, and N represents the dimensionality of the final embedding.

#### 3.2.2 Heart rate encoder

As described in Section 3.1.2, the heart rate is computed for each second of the original ECG, resulting in an initial heart rate vector of size h=θ for the θ-second recordings. We note that, upon identifying beats per minute (BPM) under 60 in a 1-second ECG segment, which renders heart rate calculation unfeasible, the methodology involves averaging the heart rate value from 1 preceding and 1 subsequent data point to fill in the missing value, ensuring consistent θ data points for θ-second recordings. The *Heart Rate Encoder* is a transformer-based neural network akin to the *Inner* and *Outer Encoders*. In terms of the attention mechanism, this module employs 1 cross-attention head instead of self-attention, succeeded by a fully connected neural network (FCN). Introducing asymmetry into the attention operation via cross-attention reduces computational complexity, enhancing the module’s efficiency. Specifically, in contrast to the projection of input with dimensions M×D (as outlined in Section 3.2.1), the Q in cross-attention is a learned matrix with dimensions N×D, where N<M. The internal embeddings of this module have a dimensionality of 512 and entirely comprise only 1 block depth. Additionally, we have incorporated Fourier feature position encoding (52) for position encoding. The primary objective of this encoder is to map the original vector h into a higher-dimensional space, enhancing both the richness and quality of the feature representation, $h\in R^{\theta }\to E_{h}\in R^{2048}$, where $E_{h}$ is the the output embedding of this encoder.

In the subsequent phase, the embedding from the heart rate encoder undergoes dimensional expansion through a bicubic interpolation module. This step generates a feature representation of the original heart rate, facilitating its seamless integration with the embedding representation of the video through the addition operation. The interpolation module underlines the necessity for identical dimensions in both embedding vectors. Importantly, this non-learning-based method proves efficient and effective for encoding. Moreover, the interpolation-based approach offers the flexibility to dynamically determine the dimensionality of the final output embedding, contrasting with the predetermined nature of a neural network-based approach. Specifically:

(9) $$B_{h}=\sum_{i=0}^{3}\sum_{\;j=0}^{3}a_{ij}(E_{h})\cdot (x-x_{0}(E_{h}){)}^{i}\cdot (y-y_{0}(E_{h}){)}^{j},\;$$

where $a_{ij}$ represents the interpolation coefficients, and $B_{h}$ denotes the resulting output vector obtained through bicubic interpolation. The dimension of $B_{h}$ is N, identical to $V_{D}$.

#### 3.2.3 AugmNet

*AugmNet*, inspired by recent advancements in augmentation literature (53), is a learning-based approach designed to learn augmentation patterns within the latent space. Unlike traditional methods that apply image augmentations (e.g., rotation, cropping) directly in the pixel space, *AugmNet* generically implements transformations on the embeddings. This approach eliminates the need for crafting specific transformations customized explicitly to each modality, e.g., image, signal, and text. In the proposed automatic pain assessment framework, integrating this module serves to regularize the learning process, mitigating overfitting concerns. Furthermore, these learning-based transformations corrupt the input embeddings. This strategy forces the subsequent model, particularly the *Temporal-Model*, to extract more refined and representative features, ultimately enhancing the model’s performance in the pain assessment task. Moreover, the proposed approach is modality-agnostic, functioning equivalently with embedding representations of any original modality, such as video and heart rate. The *AugmNet* method incorporates a neural network architecture, employing an encoder-decoder structure. Specifically, the encoder and decoder are composed of only 2 fully connected layers, with the nonlinear activation function *ELU* applied after each layer.

For a session of duration θ seconds, it results in θ×framespersecond frames and θ×samplingfrequency data points for each video and ECG, respectively. In the video analysis pipeline, the *Spatial-Module* generates an embedding representation, $V_{D}$ (8), from the original video, with dimensions d×FPS=N. In the ECG analysis pipeline, following heart rate extraction, a feature representation with a dimension of θ is produced, corresponding to one data point per second. Subsequent application of the *Heart Rate Encoder* and bicubic interpolation yields an embedding representation, $B_{h}$ (9), with dimension N. The fusion of video and heart rate embeddings occurs at a session level. Specifically, $V_{D}$ and $B_{h}$ are combined through addition, merging the information from the original input modalities. This composite embedding is subsequently fed into *AugmNet*


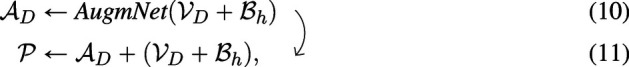


where P is the transformed embedding vector, serving as input for the final module, the *Temporal-Module*. *AugmNet* functions exclusively during the training phase as a conventional augmentation method. However, it is inactive during inference.

#### 3.2.4 Temporal-module

This module, similar to *Heart Rate Encoder*, is a transformer-based model that extends beyond multi-head cross-attention by combining it with multi-head self-attention. It features 1 multi-head cross-attention block and 3 successive multi-head self-attention blocks, with 1 and 8 attention heads correspondingly. Subsequent to each attention block, there is an FCN. Moreover, the internal embeddings within this module possess a dimensionality of 128, with a total block depth of 1. The position encoding approach is identical to the *Heart Rate Encoder*, incorporating Fourier feature position encoding. The module processes the input embedding P or the $\left(V_{D}+B_{h}\right)$ in the case that *AugmNet* is not applied, leading to the estimation of the final classification score. The learning error is calculated during this stage, and the entire framework undergoes training.

### 3.3 Supplementary augmentation methods

In addition to the *AugmNet* module, which generates learning-based transformations, we have incorporated and developed supplementary augmentation techniques. The first developed augmentation technique, *Basic*, involves a combination of polarity inversion and noise insertion. This approach introduces variations and perturbations to the original input by inverting the polarity of positive and negative elements while simultaneously introducing noise. The second technique, *Masking*, involves applying masks to the embeddings. This process sets specific elements within the vectors to zero. The size of the masks is determined by random values, ranging from 10%–20% of the input embedding size, and is applied at random positions within the vectors. Both augmentation methods operate in the latent space, similar to *AugmNet*.

### 3.4 Pretraining

Before initiating the training procedure for automatic pain assessment, we conducted individual pretraining for all modules, excluding *AugmNet*. Regarding the *Spatial-Module*, we implement a two-stage pretraining process. In the initial stage, the model undergoes pretraining on the *VGGFace2* (54), a facial recognition dataset, learning foundational facial features. Subsequently, the pretrained model undergoes optimized training with emotion recognition datasets in a multi-task learning setting. These datasets include the publicly available *AffectNet* (55), *Compound Facial Expressions of Emotions Database* (56), *RAF Face Database basic* (57), and *RAF Face Database compound* (57). This approach enables the model to learn more specific features related to emotional expressions associated with the manifestation of pain. Following the multi-task learning process, the model learns from the four datasets simultaneously. We follow the approach proposed in (58) for the multi-task learning loss, where learned weights multiply the independent losses, taking into account the homoscedastic uncertainty of each task:

(12) $$L_{\mathrm{total}}=[e^{w1}L_{S_{1}}+w_{1}]+[e^{w2}L_{S_{2}}+w_{2}]+[e^{w3}L_{S_{3}}+w_{3}]+[e^{w4}L_{S_{4}}+w_{4}],\;$$

where *L*_*S*_ is the loss for each task corresponding to different datasets and w represents the learned weights that guide the learning process to minimize the combined loss *L*_*total*_, considering all the individual losses in a balanced manner. The *Temporal-Module* is exclusively trained on the *VGGFace2* dataset. Due to its architecture, the images are first flattened into 1D vectors before fed into the module. Finally, the *Heart Rate Encoder* undergoes pretraining using the *ECG Heartbeat Categorization Dataset* (59). The specific dataset comprises two collections of heartbeat signals derived from two notable datasets in heartbeat classification: the *MIT-BIH Arrhythmia Dataset* (60) and the *PTB Diagnostic ECG Database* (61, 62). Table 1 details the datasets used in the training procedure.

**Table 1:** Publicly available datasets utilized for the pretraining process of the framework.

Dataset	# samples	# classes	Task
*VGGFace2* (54)	3.31M	9,131	Face
*AffectNet* (55)	0.40M	8	Emotion
*Compound FEE-DB* (56)	6,000	26	Emotion
*RAF-DB basic* (57)	15,000	7	Emotion
*RAF-DB compound* (57)	4,000	11	Emotion
*ECG HBC Dataset* (59)	0.45M	5	Arrhythmia

Task, all tasks involve classification.

### 3.5 Dataset details

In this research, in order to evaluate the proposed framework, the publicly accessible *BioVid Heat Pain Database* (29) wasemployed, which comprises facial videos, electrocardiograms, electromyograms, and skin conductance levels from 87 healthy participants (44 males and 43 females, aged 20–65). The dataset’s experimental design utilized a thermode to induce pain in the participants’ right arm. Before commencing data collection, the pain threshold (where sensation shifts from heat to pain) and tolerance threshold (the point at which pain becomes intolerable) of each participant were established. These thresholds defined the minimum and maximum pain levels, with two additional levels in between, resulting in four distinct pain intensities. Consequently, five intensity levels were identified: No Pain (NP), Mild Pain (P_1_), Moderate Pain (P_2_), Severe Pain (P_3_), and Very Severe Pain (P_4_). The temperatures for pain stimulation were uniformly distributed within the range from P_1_ to P_4_, never surpassing $50.5^{\circ }C$. Each participant underwent pain stimulation 20 times at the four predetermined intensity levels (P_1_ to P_4_). The application of each stimulus lasted 4 s, followed by a random recovery period between 8 to 12 s. Alongside 20 baseline measurements (NP=$32^{\circ }C$), this resulted in 100 stimulations per participant, administered in a randomized order. The dataset underwent preprocessing to segment 5.5-s windows, beginning 1-s post-reaching the target temperature for every stimulation. Consequently, it comprised 8,700 samples, each 5.5 s long, across 87 subjects, with an even distribution among the five classes for each modality.

## 4 Experimental settings & results

The study utilized the videos and electrocardiograms from Part A of *BioVid*, incorporating all available samples from the 87 subjects. The videos have a frame rate of 25 frames per second (FPS), and the ECG recordings are sampled at 512 Hz. Each session lasts 5.5 s, resulting in 138 video frames and ECG vectors with 2,816 elements, subsequently transformed into heart rate vectors of 5 data points. All the available frames and data points from videos and cardiac signals were employed in the conducted experiments. Our experimental strategy involves iteratively refining techniques and selecting the most effective combination in each round. The selected combination undergoes an extended training period (500 to 800 epochs) to enhance feature learning and potentially achieve better performance. Table 2 includes the training details of the framework regarding the automatic pain assessment task.

**Table 2:** Training details for the automatic pain assessment.

Optimizer	Learning rate	LR decay	Weight decay	Warmup epochs	Batch size
*AdamW*	$1\times 10^{-4}$	*cosine*	0.1	50	32

The pain assessment experiments were conducted in binary and multi-level classification scenarios, encompassing the evaluation of each modality separately and their combination. The binary task distinguishes between No Pain (NP) and Very Severe Pain (P_4_), while the multi-level classification (MC) involves all pain classes in the dataset. The evaluation methodology employed is the leave-one-subject-out (LOSO) cross-validation. The classification metrics include accuracy, precision, recall (sensitivity), and F1 score. In addition, it is important to note that an identical training process is maintained for both binary (NP vs. P_4_) and multi-level (MC) tasks without introducing any varying schedule or optimization.

### 4.1 Video modality

The experiments concerning the video modality include analysis into the pretraining impact of the *Spatial-Module*, the influence on the performance of the video analysis pipeline, i.e., specifically, the division into tiles, and the application of the introduced augmentation methods. Table 3 presents all the conducted experiments utilizing the video modality.

**Table 3:** Classification results utilizing the video modality reported on accuracy %.

Epochs	Pretraining stage		Pipeline		Augmentations			Task	
	1st	2nd	Full frame	Tiles	Basic	Mask	AugmNet	NP vs. P_4_	MC
500 500	✓ –	– ✓	✓ ✓	– –	✓ ✓	– –	– –	72.56 74.25	31.22 33.34
500 500 500	– – –	✓ ✓ ✓	– ✓ ✓	✓ ✓ ✓${✓}^{c}$	✓ ✓ ✓	– – –	– – –	68.07 65.11 74.86	31.49 27.84 33.86
500 500 500	– – –	✓ ✓ ✓	✓ ✓ ✓	✓${✓}^{c}$ ✓${✓}^{c}$ ✓${✓}^{c}$	✓ ✓ ✓	✓ – ✓	– ✓ ✓	73.05 74.83 73.16	32.14 33.73 32.87
800	–	✓	✓	✓${✓}^{c}$	✓	✓	✓	**77.10**	**35.39**

Stage, referring to pretraining process for *Spatial-Module*; Mask, Masking; c, constant-coefficient applied exclusively to the tiles; NP, no pain; P_4_, very severe pain; MC, multiclass pain level.

The bold values indicate the higher performance.

A noticeable difference in performance is apparent in examining the classification outcomes based on the first and second pretraining stages for the *Spatial-Module*. Specifically, when focusing on the NP vs. P_4_ task, reliance solely on the first pretraining stage yields a performance level of 72.56%. In contrast, incorporating the second stage increases the performance to 74.25%. Likewise, the difference between the second and first stages remains noteworthy in multi-level classification. The improvement is discernible, corresponding to 33.34%, with an additional 1.12% in this multi-level pain assessment task, demonstrating that the additional affective-related pretraining resulted in better embedding representations.

The subsequent series of experiments focuses on the incorporation of tiles. Initially, employing the four tiles as the frame representation resulted in a substantial decrease in performance. Specifically, there was a reduction of over 6% in the binary task and a smaller yet significant decrease of 1.85% in the multi-level task. This suggests a clear detriment to video analysis when utilizing tiles. We attribute this decline to the localized nature of each tile. The embeddings extracted from individual tiles may capture information unrelated to pain manifestation, such as unexpressed face regions or the inclusion of background elements in some frames. Subsequently, introducing the resized (i.e., 224×224) full-frame in combination with the tiles further diminished the results, yielding 65.11% and 27.84% accuracy for the binary and multi-level tasks, respectively. Despite the unfavorable outcome, incorporating the full-frame was considered valuable since the initial experiments were based on achieving promising results. This drove the subsequent experiment, where the full-frame was combined with the tiles, introducing a coefficient applied to the latter. The introduction of a coefficient (c=0.1) involves multiplying the tile embeddings, retaining only 10% of the initial information. This modification enhanced performance, achieving 74.86% and 33.86% accuracy for the tasks, showcasing an improvement of 0.61% and 0.52% compared to the exclusive utilization of the full-frame.

Two augmentation methods, *Masking* and *AugmNet*, were introduced in the subsequent experiments alongside *Basic*. The application of *Masking* resulted in a performance decline of 1.81% and 1.72%, while *AugmNet* also contributed to a decrease, albeit smaller, with a reduction of 0.03% and 0.13%. Applying both methods yielded accuracy levels that surpassed those achieved with *Masking* isolated but did not reach the individual performance observed with *AugmNet*. Despite the immediate outcomes, combining all augmentation methods emerged as the most promising choice for prolonged training. This decision stems from the consideration that an extended training period may introduce overfitting concerns. A heavy regularization strategy, exemplified by the combined application of all augmentation methods, is anticipated to address and mitigate potential overfitting issues effectively. Indeed, the prolonged training period resulted in final accuracy rates of 77.10% and 35.39% for binary and multi-level pain classification, respectively, in the unimodal vision-based approach.

### 4.2 Heart rate modality

The experiments regarding the heart rate modality include the utilization of the encoder and the application of the introduced augmentation methods. Table 4 presents all the conducted experiments utilizing the heart rate modality.

**Table 4:** Classification results utilizing the heart rate modality reported on accuracy %.

Epochs	HR encoder	Augmentations			Task	
		Basic	Mask	AugmNet	NP vs. P_4_	MC
500 500	– ✓	✓ ✓	– –	– –	61.70 61.93	27.60 27.68
500 500 500	✓ ✓ ✓	✓ ✓ ✓	✓ – ✓	– ✓ ✓	61.95 62.09 61.87	27.73 28.11 27.96
800 800	✓ ✓	✓ –	✓ –	✓ ✓	64.84 **67.04**	29.81 **31.22**

The bold values indicate the higher performance.

Employing the original heart rate vectors with a dimensionality of h=5, the classification scores for distinguishing between NP and P_4_ were 61.70% and for the multi-level task 27.60%. Subsequently, through the application of the *Heart Rate Encoder*, which mapped the original vectors to a higher-dimensional space, resulting in embeddings of size h=2048, there was a modest enhancement in performance. Specifically, we observed an increment of 0.23% for the binary classification task and 0.08% for the multi-level classification task. The observed performance increase appears minimal despite the expanded representation of the encoded heart rate vector due to its increased size. This phenomenon may be attributed to the fact that, although the embedding is over 400% larger than the original input, the inherent information within the limited data points defining the heart rate may not be sufficient to yield a significantly improved feature representation. Despite the marginal improvement, the utilization of the encoder remains crucial as our objective is to generate larger-sized embeddings. This is particularly essential for our multimodal approach, where we seek to integrate information from video and heart rate data. Further details on this integration will be expounded upon in the following section.

Similar to the video modality, experiments regarding the augmentation methods applied to the heart rate were conducted. The application of *Masking* resulted in an increase of 0.02% for the binary task and 0.05% for the multi-level task. Correspondingly, *AugmNet* led to further improvement, reaching 62.09% and 28.11% for the binary and the multi-level tasks, respectively, while combining all the augmentation methods led to a decrease, resulting in 61.87% and 27.96%. During the extended training period of 800 epochs, a classification accuracy of 64.87% was attained for the binary task and 29.81% for the multi-level task when employing a combination of all augmentation methods. Despite the increase, we observed that introducing augmentations to the heart rate signal poses more challenges for accurate classification than the video. For this reason, we conducted a repeat of the extended training experiment. In this iteration, we excluded the augmentation methods of *Basic* and *Masking*, retaining only *AugmNet*. As a result, the performance for the binary task improved to 67.04%, and for the multi-level task, it reached 31.22%. Reducing corruption within the heart rate embedding space contributed to enhanced performance. We observe a moderate divergence in the behavior of the augmentation pipeline between the heart rate and video modality. This indicates the challenges of employing a single extracted isolated feature as input in a machine learning-based system. We hypothesize that the limited information encapsulated in heart rate embeddings makes them more susceptible to significant degradation caused by augmentations. This stands in contrast to video embeddings, derived from a more extensive and information-rich modality, which can more gracefully accommodate such augmentations.

### 4.3 Multimodality

The results for the fusion of the two modalities are presented in Table 5. Drawing insights from the experiments conducted on video and heart rate modalities, we opted for an extended training time of 800 epochs. Utilizing the tiles with coefficient c=0.1, *AugmNet* was exclusively employed as the augmentation method. The proposed strategy achieved a classification accuracy 82.74% for NP vs. P_4_, while for the multi-level classification task, it reached 39.77%. This represents a notable improvement, with a 5.64% and 15.70% higher performance compared to the video and heart rate modalities, respectively, for the binary task. Similarly, the combined approach demonstrates a 4.38% and 8.55% performance increase for the multi-level task over the individual modalities. Integrating these two crucial modalities yields highly effective pain assessment performances and surpasses the results achieved by each modality.

**Table 5:** Classification results utilizing the video & the heart rate modality reported on accuracy %.

Epochs	HR encoder	Pipeline		Augmentations			Task	
		Full frame	Tiles	Basic	Mask	AugmNet	NP vs. P_4_	MC
800	✓	✓	✓^*c*^	–	–	✓	**82.74**	**39.77**

The bold values indicate the higher performance.

### 4.4 Comparison with existing methods

In this section, we conduct a comparative analysis of the results of our method with other existing approaches in the literature. Our evaluation utilizes Part A of the *BioVid* dataset, involving all 87 subjects. It follows the same evaluation protocol—leave-one-subject-out (LOSO) cross-validation—to ensure objective and accurate comparisons. Specifically, we compare our approach with both unimodal and multimodal studies, categorizing them into (1) video-based studies, (2) ECG-based studies, and (3) multimodal studies, irrespective of the number or type of modalities employed. The corresponding results are summarized in Table 6.

**Table 6:** Comparison of studies utilizing *BioVid* & LOSO cross-validation reported on accuracy %.

Study	Modality	Method				Task	
		Features	Machine learning	Params (M)	FLOPS (G)	NP vs. P_4_	MC
(30)	Video	Optical flow	RF	–	–	70.20	–
(31)	Video	Raw	SLSTM	–	–	61.70	29.70
(32)	Video	Raw	2D CNN, 3D CNN	423.20	–	86.02	–
(33)	Video	Raw	3D CNN,	–	–	77.50	34.30
(34)	Video	Raw	2D CNN, biLSTM	–	–	69.25	–
(35)	Video	Raw	2D CNN	25.00^⊚^	4.00	71.00	–
(36)	Video	Facial action descriptors	Deep RF	–	–	72.40	30.80
(36)	Video	Facial 3D distances	Deep RF	–	–	72.10	30.30
(37)	Video	Fiducial points	GNN	–	–	73.20	–
(38)	Video	Raw	2D CNN, AE-ATT	–	–	**86.65**	**40.40**
(39)^†^	Video	Raw	2D CNN	–	–	71.30	37.60
(40)^†^	Video	Raw	2D CNN, GRU	150.00^⊚^	–	73.90	39.10
(41)	Video	Raw	Transformer	24.00	4.20	73.28	31.52
(42)	Video	Facial landmarks, 3D distances	RF			71.60	–
Our	Video	Raw	Transformer	4.20^⊛^	1.62	77.10	35.39
(25)	ECG	Raw	1D CNN	1.80^⊚^	–	57.04	23.23
(27)	ECG	Domain-specific^⋇^	LR	–	–	57.69	–
(28)	ECG	Domain-specific^⋇^	SVM	–	–	58.39	23.79
(33)	ECG	Heart rate^★^	3D CNN	–	–	65.00	28.50
(42)	ECG	Domain-specific	RF	–	–	62.00	–
(43)	ECG	Domain-specific	FCN	4.09^⊙^	0.40	**69.40**	30.24
(44)	ECG	Domain-specific^⋇^	SVM	–	–	63.50	–
Our	ECG	Heart rate	Transformer	6.03^⊛^	1.25	67.04	31.22
(25)	ECG, EMG, GSR	Raw	2D CNN	10.00^⊚^	–	76.72	36.54
(27)	ECG, GSR	Domain-specific^⋇^	SVM	–	–	72.20	–
(33)	Video${\;}^{1}$, ECG${\;}^{2}$	Raw${\;}^{1}$, heart rate${\;}^{2}$^★^	3D CNN	–	–	**88.10**	**42.20**
(42)	ECG${\;}^{1}$, EMG${\;}^{1}$, GSR${\;}^{1}$	Domain-specific${\;}^{1}$^⋇^	RF	–	–	74.10	–
(42)	Video${\;}^{1}$, ECG${\;}^{2}$, EMG${\;}^{2}$, GSR${\;}^{2}$	Facial landmarks${\;}^{1}$, 3D distances${\;}^{1}$, domain-specific${\;}^{2}$^⋇^	RF	–	–	77.80	–
(44)	Video${\;}^{1}$, ECG${\;}^{2}$, GSR${\;}^{2}$	Facial landmarks${\;}^{1}$, 3D distances${\;}^{1}$, domain-specific${\;}^{2}$^⋇^	RF	–	–	78.90	–
(44)	Video${\;}^{1}$, ECG${\;}^{2}$, EMG${\;}^{2}$, GSR${\;}^{2}$	Facial landmarks${\;}^{1}$, 3D distances${\;}^{1}$, domain-specific${\;}^{2}$^⋇^	SVM	–	–	76.60	–
(47)	ECG, EMG, GSR	Raw	DDCAE	4.00^⊚^	–	83.99	–
Our	Video${\;}^{1}$, ECG${\;}^{2}$	Raw${\;}^{1}$, heart rate${\;}^{2}$	Transformer	8.60^⊛^	2.44	82.74	39.77

M, millions; G, Giga; RF, random forest; AE-ATT, autoencoder attention; SVM, support vector machines; LR, logistic regression; –, missing value.

The bold values indicate the higher performance.

^†^ Reimplemented for pain intensity estimation on *BioVid* by (33).

^★^ Pseudo heart rate gain.

^⋇^ Numerous features.

^⊚^ Parameter count estimated from provided paper details.

^⊛^ *AugmNet* excluded from parameter count, not used in inference.

^⊙^ Parameter count not mentioned in study, provided directly by authors.

In video-based studies, our method, achieving 77.10% for the binary task and 35.39% for the multi-level task, stands out as one of the most effective in terms of performance. Notably, it surpasses the average performance of the other studies by approximately 4.7% for the binary and 3.4% for the multi-level pain assessment. In the context of ECG-based studies, our method demonstrated noteworthy performance with 8.5% and 18.1% higher accuracy than the average for the binary and multi-level tasks, respectively. Intriguingly, our approach achieved the highest classification performance for the multi-level task, reaching 31.22%. These results are particularly significant as our method solely utilizes the heart rate as an extracted feature from the electrocardiography. This showcases not only its capability to assess pain but also its ability to attain state-of-the-art results. Finally, in multimodal studies, our method achieved a noteworthy accuracy of 82.74% for the NP vs. P_4_ task, placing it among the top results. It is outperformed only by studies (33, 47), which reported 88.10% and 83.99%, respectively. For the multi-level task, direct comparisons are limited as only a few studies have conducted this specific experiment. Study (33) achieved 42.20%, and (25) reported 36.54%, highlighting the competitive performance of our method in this context.

### 4.5 Inference time

**Figure 3:**
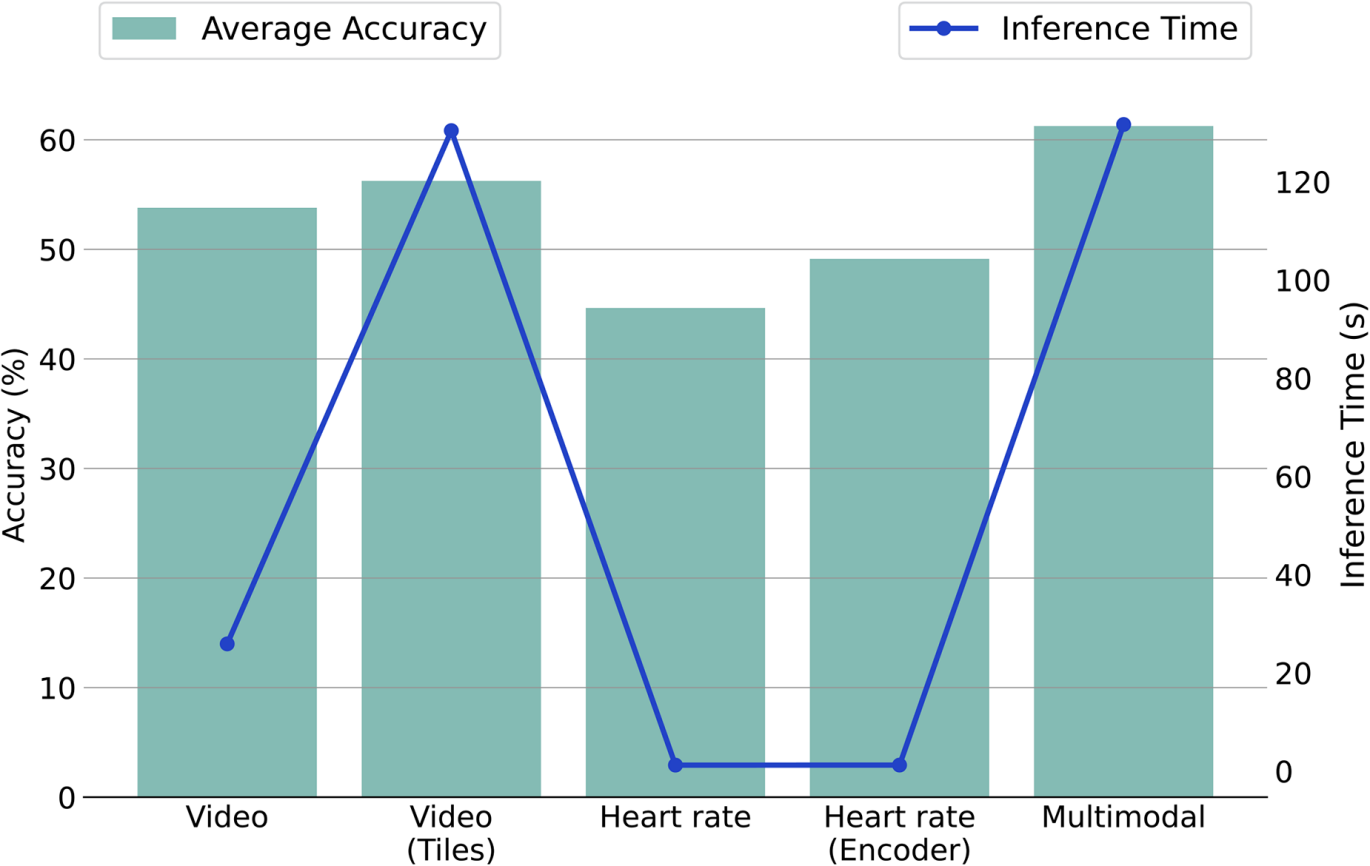
Comparison of average accuracy and inference time for unimodal and multimodal methodologies across NP vs. P_4_ and MC tasks. Note: The plot employs a dual-*y*-axis format (left for accuracy, right for time) to illustrate the relation between performance and efficiency, with methodologies listed on the *x*-axis.

We investigated the video-based approach, the video with the additional usage of tiles, the heart rate-based approach, the heart rate concurrently with the encoder, and the multimodal approach. Figure 3 represents each method’s inference time in seconds (s) and the corresponding average accuracy performances between the binary and multi-level tasks. Table 7 outlines the number of parameters and the computational cost in terms of floating-point operations (FLOPS) for each component. The inference times are derived on an *Intel Core i7-8750H* CPU. The reported inference time encompasses face detection for each frame but excludes the heart rate extraction from the original electrocardiography. This intentional exclusion aligns with our focus on investigating the usage of heart rate as a cardiac feature automatically provided by wearables.

**Table 7:** Number of parameters and FLOPS for the components of the proposed framework.

Module	Params (M)	FLOPS (G)
Spatial-module	2.57	1.19
Heart rate encoder	4.40	0.82
AugmNet	1.02	0.02
Temporal-module	1.63	0.43
Total	9.62	2.46

We observe that the inference time for the video modality using the original pipeline is approximately 26 s. However, adopting the tile pipeline increases the inference time dramatically to about 130 s. The time increase is expected, considering that in the first case, a single image representation is used for each frame, while in the second case, five image representations are employed (one full frame and four tiles). In the heart rate signal context, 1.2 s are required for the completion of a pain assessment. Notably, with the integration of the *Heart Rate Encoder*, the processing time remains nearly unchanged, showing a slight increase of less than half a second. This underscores the efficiency inherent in this specific module. Finally, the proposed multimodal framework, incorporating the tiles and the *Heart Rate Encoder*, requires about 131 s.

### 4.6 Interpretation

**Figure 4:**
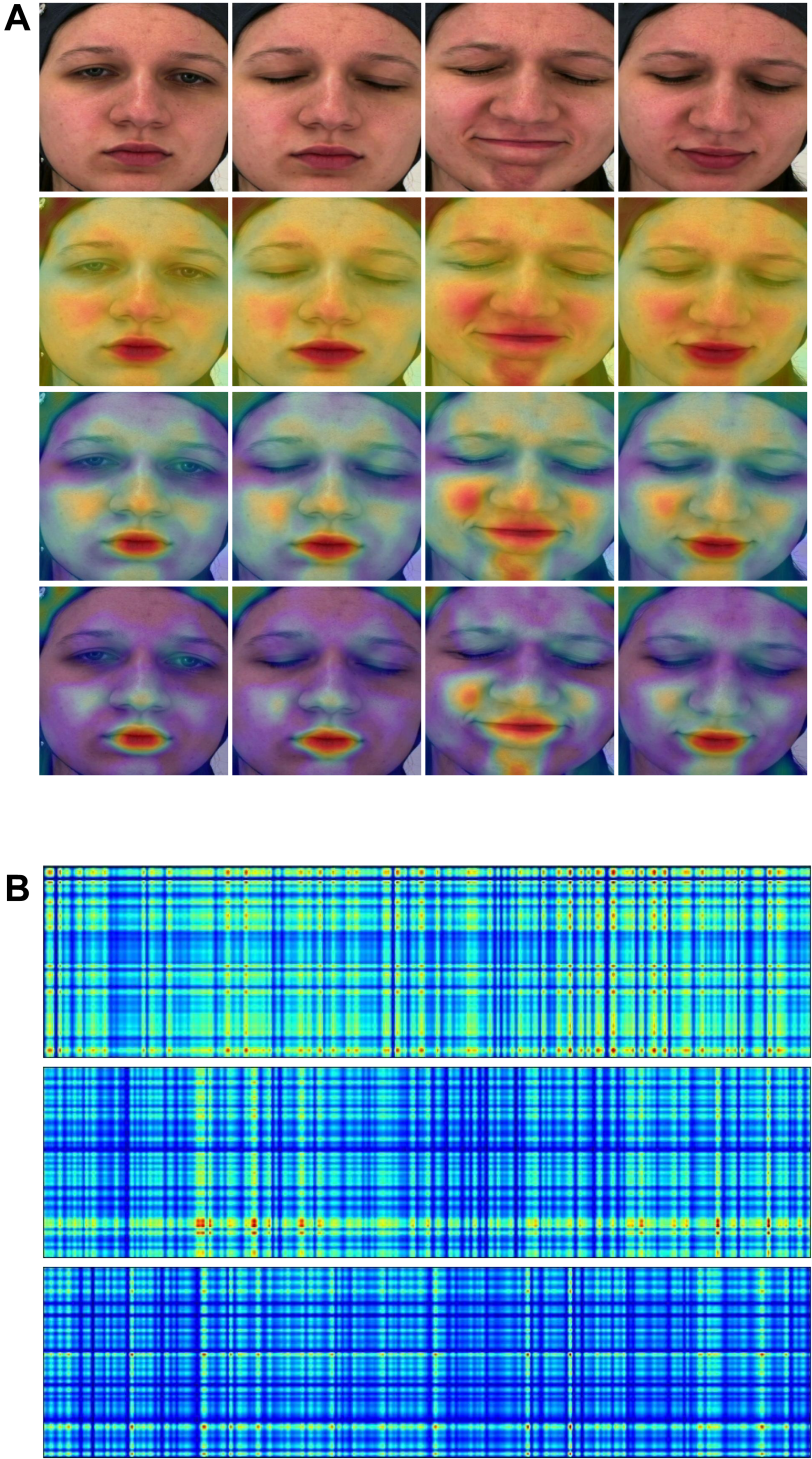
(A) Attention maps from the Spatial-Module. (B) Attention maps from the Temporal-Module. Yellow and red colors indicate high attention to the particular region. (**A**) **(1st row)** Original frame sequence. **(2nd row)** Computed from the *Spatial-Module* following the first stage pretraining. **(3rd row)** Computed from the *Spatial-Module* following the second stage pretraining. **(4th row)** Computed from the *Spatial-Module* trained on *BioVid*. (**B**) **(1st row)** Computed from the *Temporal-Module* with video embedding. **(2nd row)** Computed from the *Temporal-Module* with heart rate embedding. **(3rd row)** Computed from the *Temporal-Module* with fused (video & heart rate) embedding.

Improving the models’ interpretability is crucial for gaining acceptance and integrating them effectively into the clinical domain. In this study, attention maps have been generated from both the *Spatial-Module* and the *Temporal-Module* (examples are illustrated in Figure 4).

Concerning the *Spatial-Module*, attention maps are generated based on the weights contribution of the last fully connected layer. These weights are then interpolated onto the images, effectively visualizing the areas where the model focuses. In Figure 4A, we present an original frame sequence alongside three variations of attention maps: (1) derived when the *Spatial-Module* followed the first stage of pretraining, (2) after the second stage of pretraining, and (3) when trained on *BioVid*. In the first pretraining stage, i.e., founded on a face recognition task, the *Spatial-Module* generates attention maps focusing on the entire facial region, emphasizing specific areas. The model exhibits heightened attention to facial components, emphasizing the zygomatic and buccal regions as well as the oral and mental regions. Furthermore, we observe a discernible focus on the nasal region. In the second pretraining stage, founded on a multi-task emotion recognition setting, the *Spatial-Module* produces attention maps with heightened specificity. In contrast to the first pretraining stage, where attention is distributed across the entire face region, the model focuses on distinct areas. These areas coincide with those emphasized in the first pretraining stage but exhibit more pronounced and explicit attention. Furthermore, attention maps generated after training on the *BioVid* dataset for pain assessment reveal additionally heightened explicitness towards distinct facial areas. The identified areas of interest persist across all three variations of attention maps. The notable distinction lies in the decreased attention in less relevant regions, directing and maintaining focus on the critical areas. Finally, as pain-related expressions manifest, the attention maps consistently depict the model’s adeptness at recognizing these variations and directing its focus accordingly.

Attention maps were also generated from the *Temporal-Module* utilizing input embeddings. These maps are also derived from the contribution of weights in the module’s final fully connected layer and interpolated alongside the input, creating rectangle shapes which are easy to visualize. Figure 4B provides an example with three distinct input scenarios: (1) a video embedding, (2) a heart rate embedding, and (3) a fused embedding, combining video and heart rate. Overall, we observed that the attention maps in all three versions exhibit a grid-like pattern reminiscent of a tartan design, showcasing varying spatial frequencies that seem to scan the input. This phenomenon may be attributed to incorporating Fourier position encoding, as mentioned in (52), in the context of similar perceiver-like transformer architectures. The attention map generated from the video embedding input reveals a high overall intensity of attention across the input. On the other hand, the heart rate input generates a map with less attention spread across the input, yet there are specific areas marked by a notably high focus indicated by the red color. The attention map from the fused embedding demonstrates a medium intensity, aligning with expectations due to the combination of video and heart rate embeddings. We observe a tendency in the attention maps, particularly those generated from the video and fused embeddings, to exhibit a pronounced focus on the right portion of the rectangle, corresponding to the session’s conclusion. This aligns with the real-time manifestation of pain experienced by the subject towards the end of the session.

## 5 Discussion

In this study, we developed a multimodal framework that leverages videos and heart rate signals for automatic pain assessment. Our proposed method comprises four pivotal modules distinguished by efficacy and efficiency. Notably, boasting a mere 2.57 million parameters, the *Spatial Module* stands out as one of the small-scale and most efficient vision-based models of automatic pain assessment documented in the literature. In our case, given the limited studies available or from which we could extract relevant information, it has been demonstrated that our proposed model is capable of achieving comparable or superior performance with a significantly smaller model size. Indeed, our model’s efficiency and high performance are attributed to the specific module’s rigorous and sophisticated pretraining process. We presented the substantial benefits of multi-task pretraining on affective-related datasets, a key factor enabling the development of an efficient model with exceptional performance in the downstream task of pain estimation. The highly efficient *Heart Rate Encoder* module, comprising 4.40 million parameters, demonstrated remarkable effectiveness in encoding the original heart rate vector into a higher-dimensional space for fusion with the video embedding. During inference, the entire encoding process is completed in less than half a second. This efficiency is attributed to the combination with the applied bicubic interpolation, which facilitates dynamic encoding of inputs and allows for generating outputs with arbitrary sizes on the fly. The *AugmNet* module is a learning-based augmentation method that generates transformations within the latent space. This eliminates the necessity to craft specific design augmentations for each modality. It is important to note that, akin to other augmentation techniques, careful consideration is required in their application to prevent issues such as over-regularization or other challenges related to the learning process of the models. The *Temporal-Module*, featuring a modest 1.63 million parameters, serves as the final component responsible for estimating the pain level of a session. It utilizes embeddings derived from either video, heart rate, or a combination. A notable characteristic of this module is its integration of cross- and self-attention, contributing to its efficacy and efficiency. All the modules utilized in inference, except *AugmNet*, were founded on the transformer architecture. This underscores that adequate pretraining and optimization can achieve compelling results with a minimal model size. This is particularly noteworthy as transformer-based models are often associated with large-scale settings, and our approach showcases their efficacy even in compact configurations.

Our experiments demonstrated that video can be a valuable source of information for understanding an individual’s experience of pain. This holds because a video captures various aspects of a person’s behavior, such as facial expressions, eye gaze, head movements, and even subtle changes in skin color during stressful experiences such as painful events. Our approach achieved an accuracy of 77.10% for the binary classification task, distinguishing between no pain and very severe pain by leveraging the video modality. Additionally, for the multi-level pain classification task, encompassing five pain levels, including the no-pain condition, we attained a recognition accuracy of 35.39%. Our exploration of the heart rate signal, as a proof of concept, demonstrated that outstanding results can be achieved even with this singular feature extracted from electrocardiography. This carries significant importance, aligning with our primary objective of investigating the potential utility of this particular feature, considering its automatic availability from nearly every wearable device in the market. Notably, this eliminates the need for explicitly designing algorithms or systems for calculating cardiac features or utilizing raw biosignals, saving time and computational resources. Based exclusively on the heart rate, our method achieved an accuracy of 67.04% for the binary task and an impressive 31.22% for the multi-level task, surpassing the best performance reported in the literature. This underscores the feasibility and the exceptional performance offered by the exclusive adoption of heart rate as a predictive feature. The multimodal approach we proposed, fusing video and heart rate modalities, showcased impressive results. Achieving 82.74% and 39.77% for the binary and multi-level tasks, respectively, surpasses the effectiveness of the video modality by approximately 9% and the heart rate modality by about 24%. In addition, these results position it as one of the leading performances in the literature, with only 9.62 million parameters in total. This highlights the efficacy of a thoughtfully designed system that combines two modalities, demonstrating superior performance compared to each modality in isolation.

In interpreting our framework, attention maps from the *Spatial-Module* highlighted important facial areas, such as the zygomatic and oral regions, contributing significantly to automatic pain assessment. Different pretraining stages impacted these maps, revealing more explicit attention with specialized training. Attention maps from the *Temporal-Module* showed a subtle focus on the last part of the input, where pain manifestations typically appear in the particular dataset.

Throughout this study, we delved into the potential utility of video and heart rate modalities for pain assessment. Furthermore, we demonstrated the advantages of adopting a multimodal approach, leveraging the strengths of both modalities. It is essential to highlight that our experiments utilized the only publicly available dataset designed explicitly for pain assessment, encompassing both facial videos and cardiac signals, created under controlled laboratory conditions. Participants were seated in a frontal position, benefiting from optimal lighting conditions for video recordings, and physiological sensors were meticulously attached to the body. However, it is crucial to acknowledge that challenges may arise in real-world scenarios, particularly in clinical environments. Factors such as variations in lighting, unpredictable facial positioning, facial occlusions, or sensor attachment difficulties require careful consideration and optimization when developing systems for such practical applications. Additionally, the reliance on heart rate as the sole cardiac feature may face limitations in challenging environments, underscoring the need for a combination of extracted features or the utilization of raw biosignals.

## 6 Conclusions

This study explored the effectiveness of facial video and heart rate in automatic pain assessment and analyzed the advantages and limitations of each modality. The experiments, along with direct comparisons to 14 video-based, 7 ECG-based, and 8 multimodal-based studies, substantiated the efficacy of the proposed framework, delivering high classification results while maintaining outstanding efficiency. In the multimodal setting, the framework achieved 82.74% and 39.77/% accuracy for the binary and multi-level pain assessment tasks, respectively, with less than 10 million total parameters. Given appropriate optimization for practical applications, we believe that such a framework holds promise for real-world scenarios. Moreover, by generating attention maps, we provided insights into the functioning of specific modules by revealing the focus areas within the inputs. The proposed framework, characterized by high efficiency, has the potential to achieve even better performances through the scaling up of individual modules. However, this enhancement comes at the cost of reduced efficiency and speed, a trade-off that should be carefully considered based on specific application requirements. Furthermore, researchers are encouraged to provide details regarding the computational costs of their approaches. This transparency would be valuable for other researchers, facilitating comparisons and offering insights into the computational efficiency of different methodologies. We recommend that future studies utilize multi-modalities, as it is the most effective approach for assessing the pain phenomenon in real-world settings. Developing interpretation methods is also crucial, particularly for the prospective integration of these frameworks into clinical practice.

## Data availability statement

Publicly available datasets were analyzed in this study. This data can be found here: https://www.nit.ovgu.de/nit/en/BioVid-p-1358.html.

## Ethics statement

Written informed consent for the publication of any potentially identifiable images or data included in this article was not required as the images used in this study originate from publicly available datasets.

## Figures and Tables

**Figure A1:**
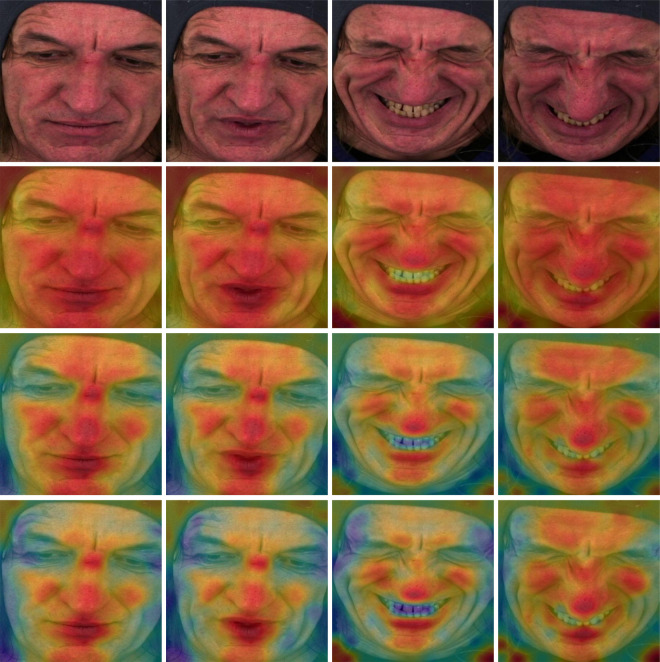
Attention maps from the *Spatial-Module*. Yellow and red color indicates high attention to the particular region. **(1st row)** Original frame sequence. **(2nd row)** Computed from the *Spatial-Module* following the first stage pretraining. **(3rd row)** Computed from the *Spatial-Module* following the second stage pretraining. **(4th row)** Computed from the *Spatial-Module* trained on *BioVid*.

**Figure A2:**
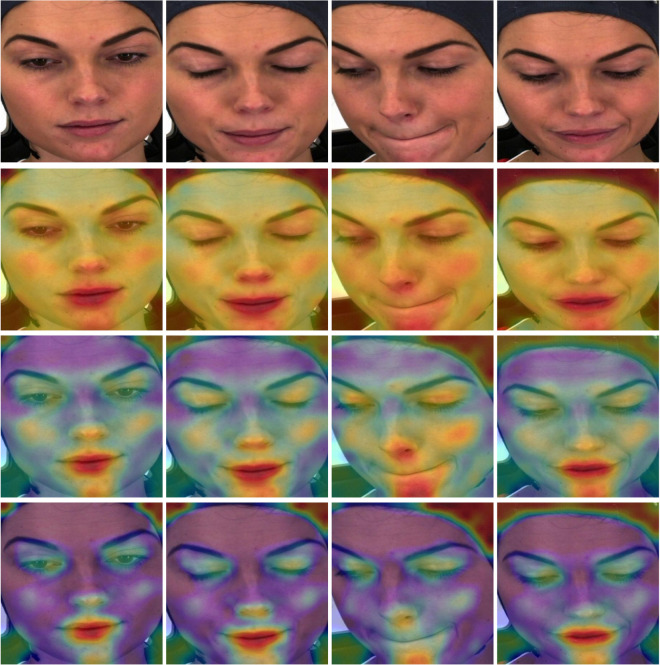
Attention maps from the *Spatial-Module*. Yellow and red color indicates high attention to the particular region. **(1st row)** Original frame sequence. **(2nd row)** Computed from the *Spatial-Module* following the first stage pretraining. **(3rd row)** Computed from the *Spatial-Module* following the second stage pretraining. **(4th row)** Computed from the *Spatial-Module* trained on *BioVid*.

## Appendices

### Supplementary metrics

Table A1 presents precision, recall, and F1 score results. The second pretraining stage’s impact on the *Spatial-Module* is evident across all three metrics. Recall experiences a substantial performance boost, reaching 76.74% and 33.41% for binary and multiclass tasks, respectively. This emphasizes the profound influence of the emotion recognition pretraining process on sensitivity (i.e., identifying true positive samples) within the framework. Consistent with the accuracy findings, the incorporation of tiles negatively affects all metrics. Once again, recall is the metric most significantly impacted, dropping to 18.42%. This suggests a potential challenge in accurately identifying individuals experiencing pain (true positive instances), indicating a risk of mis-evaluation. The introduction of the coefficient (c=0.1) in tile embeddings leads to improvements in all metrics, mirroring the tendencies observed in accuracy. Similarly, the adoption of augmentation methods initially results in performance reductions. However, with extended training time, a balance between regularization and learning is achieved. This manifests in a recall of 79.35% for the binary task and a precision of 35.39% for the multiclass task.

**Table A1:** Classification results utilizing the video modality reported on precision, recall, and F1 score.

Epochs	Metric	Pretraining stage		Pipeline		Augmentations			Task	
		1st	2nd	Full frame	Tiles	Basic	Mask	AugmNet	NP vs. P_4_	MC
500	Precision	✓	–	✓	–	✓	–	–	72.53	31.24
	Recall	✓	–	✓	–	✓	–	–	74.31	29.61
	F1	✓	–	✓	–	✓	–	–	71.95	27.16
500	Precision	–	✓	✓	–	✓	–	–	74.21	33.36
	Recall	–	✓	✓	–	✓	–	–	76.74	33.41
	F1	–	✓	✓	–	✓	–	–	72.24	28.77
500	Precision	–	✓	–	✓	✓	–	–	68.11	31.50
	Recall	–	✓	–	✓	✓	–	–	72.15	27.99
	F1	–	✓	–	✓	✓	–	–	65.92	25.14
500	Precision	–	✓	✓	✓	✓	–	–	65.14	27.78
	Recall	–	✓	✓	✓	✓	–	–	70.36	18.42
	F1	–	✓	✓	✓	✓	–	–	61.93	18.86
500	Precision	–	✓	✓	✓${✓}^{c}$	✓	–	–	74.88	33.96
	Recall	–	✓	✓	✓${✓}^{c}$	✓	–	–	77.41	34.31
	F1	–	✓	✓	✓${✓}^{c}$	✓	–	–	73.90	29.20
500	Precision	–	✓	✓	✓${✓}^{c}$	✓	✓	–	73.09	32.17
	Recall	–	✓	✓	✓${✓}^{c}$	✓	✓	–	75.72	28.41
	F1	–	✓	✓	✓${✓}^{c}$	✓	✓	–	71.92	26.02
500	Precision	–	✓	✓	✓${✓}^{c}$	✓	–	✓	74.87	33.88
	Recall	–	✓	✓	✓${✓}^{c}$	✓	–	✓	77.80	29.30
	F1	–	✓	✓	✓${✓}^{c}$	✓	–	✓	73.59	27.74
500	Precision	–	✓	✓	✓${✓}^{c}$	✓	✓	✓	73.12	32.79
	Recall	–	✓	✓	✓${✓}^{c}$	✓	✓	✓	76.18	28.51
	F1	–	✓	✓	✓${✓}^{c}$	✓	✓	✓	71.91	26.57
800	Precision	–	✓	✓	✓${✓}^{c}$	✓	✓	✓	**77.15**	**35.39**
	Recall	–	✓	✓	✓${✓}^{c}$	✓	✓	✓	7**9.35**	**35.11**
	F1	–	✓	✓	✓${✓}^{c}$	✓	✓	✓	**76.33**	**31.70**

The bold values indicate the higher performance.

Table A2 presents performance metrics regarding the heart rate signal. As mentioned in the main manuscript, utilizing the *Heart Rate Encoder* improved the results, but slightly. The most significant impact was for the recall metric, which reached 66.01% and 22.13%, and improvement of 0.9% and 1.22% for the pain estimation tasks, binary and multi-level. Similar observations related to the application of augmentation methods are also evident in this context. The *AugmNet*, being a learning-based method, introduces a form of corruption to the input embedding of the heart rate, albeit in a less intrusive manner compared to the *Basic* and *Masking* methods. This makes *AugmNet* the most effective approach for this particular modality. Across all metrics, improvements were observed with the restricted augmentation pipeline. Precision demonstrated an average 2.59% increase during the extended training period, while recall and F1 scores exhibited growths of 3.42% and 4.68%, respectively.

**Table A2:** Classification results utilizing the heart rate modality reported on precision, recall, and F1 score.

Epochs	Metric	HR encoder	Augmentations			Task	
			Basic	Mask	AugmNet	NP vs. P_4_	MC
500	Precision	✓	✓	–	–	61.73	27.66
	Recall	✓	✓	–	–	65.04	20.91
	F1	✓	✓	–	–	57.74	19.73
500	Precision	–	✓	–	–	61.97	27.71
	Recall	–	✓	–	–	66.01	22.13
	F1	–	✓	–	–	57.79	20.61
500	Precision	✓	✓	✓	–	61.97	27.80
	Recall	✓	✓	✓	–	65.27	20.98
	F1	✓	✓	✓	–	57.38	20.97
500	Precision	✓	✓	–	✓	62.09	28.00
	Recall	✓	✓	–	✓	65.73	21.27
	F1	✓	✓	–	✓	58.04	21.61
500	Precision	✓	✓	✓	✓	61.63	27.86
	Recall	✓	✓	✓	✓	65.08	21.24
	F1	✓	✓	✓	✓	56.78	21.17
800	Precision	✓	✓	✓	✓	65.44	29.73
	Recall	✓	✓	✓	✓	69.85	27.40
	F1	✓	✓	✓	✓	62.07	23.71
800	Precision	✓	–	–	✓	**67.07**	**31.11**
	Recall	✓	–	–	✓	**71.24**	**29.33**
	F1	✓	–	–	✓	**63.97**	**25.83**

The bold values indicate the higher performance.

Table A3 presents the outcomes of the proposed multimodal approach. For the binary class of NP vs. P_4_, all metrics showcased performances exceeding 80%, signifying a substantial improvement compared to the unimodal video and heart rate methods. Likewise, precision, recall, and F1 scores in the multi-level classification reached 39.13%, 37.67%, and 36.31%, respectively.

**Table A3:** Classification results utilizing the video & the heart rate modality reported on precision, recall and F1 score.

Epochs	Metric	HR encoder	Pipeline		Augmentations			Task	
			Full frame	Tiles	Basic	Mask	AugmNet	NP vs. P_4_	MC
800	Precision	✓	✓	✓_c_	–	–	✓	82.69	39.13
	Recall	✓	✓	✓_c_	–	–	✓	84.71	37.67
	F1	✓	✓	✓_c_	–	–	✓	81.44	36.31

### Supplementary attention maps

As outlined in the main manuscript, the *Spatial-Module* produces three distinct variations of attention maps. An exemplar is depicted in Figure A1, highlighting a consistent pattern in the regions of interest. With specialized training, the maps become more specific. Subtle distinctions appear between the maps derived from emotion-based pretraining (3rd row) and those optimized for pain assessment (4th row). In the latter, the model exhibits reduced attention to areas like teeth and the periphery of the face. In Figure A2, a discernible contrast is evident among the three variations of attention maps. The overall focus diminishes significantly as the model undergoes training on more relevant datasets (i.e., emotion, pain), concentrating on specific regions.
